# Sublethal effects of the novel *cis*-nitromethylene neonicotinoid cycloxaprid on the cotton aphid *Aphis gossypii* Glover (Hemiptera: Aphididae)

**DOI:** 10.1038/s41598-018-27035-7

**Published:** 2018-06-11

**Authors:** Li Cui, Huizhu Yuan, Qiyuan Wang, Qinqin Wang, Changhui Rui

**Affiliations:** Key Laboratory of Integrated Pest Management in Crops, Ministry of Agriculture, Institute of Plant Protection, Chinese Academy of Agricultural Sciences, Beijing, 100193 China

## Abstract

Cycloxaprid is a novel *cis*-configuration neonicotinoid insecticide that is effective against a wide range of insect pests, including those that are resistant to conventional neonicotinoids. In this study, life table parameters were applied to estimate the cycloxaprid-induced sublethal effects on *Aphis gossypii*. The results indicated that the LC_20_ (0.81 mg a.i. L^−1^) of cycloxaprid significantly decreased the pre-oviposition period in first-progeny adults. Additionally, the life expectancy of F1 generation adults was reduced. However, no significant differences were observed for the intrinsic rate of increase (*r*_*i*_), finite rate of increase (*λ*), net reproductive rate (*R*_0_), or mean generation time (*T*) of F1 individuals. Therefore, resurgence in the *A*. *gossypii* population induced by a low concentration of cycloxaprid might not occur. Additionally, the response of the detoxification enzymes showed that cycloxaprid at the LC_20_ inhibited cytochrome P450 monooxygenase (P450) and glutathione S-transferase (GST) activities at 6 h after exposure. Such inhibition of P450 and GST activities could lead to a decrease in the metabolism of cycloxaprid, which would increase the efficacy of cycloxaprid. Therefore, our results contribute to the assessment of the overall effects of cycloxaprid on *A*. *gossypii*.

## Introduction

The cotton aphid, *Aphis gossypii* Glover (Hemiptera: Aphididae), is globally one of the most destructive sucking pests on cotton and numerous crops, causing an overall 4% reduction in lint yield annually^1^. These aphids damage plants by direct feeding, honeydew excretion and transmission of viruses, such as the virus that causes cotton bunchy top disease^2,3^. Although several control strategies have been tested and employed to suppress *A*. *gossypii*, chemical management remains the basis of Integrated Pest Management (IPM) programmes against this insect pest^1^. A diversity of chemical insecticides are used globally for controlling *A*. *gossypii*. One class, neonicotinoids, were introduced two decades ago^4^. However, the widespread use of *trans*-nitromethylene neonicotinoids such as imidacloprid has induced the development of resistance in *A*. *gossypii* worldwide. For example, Koo *et al*. reported that *A*. *gossypii* collected from Korea exhibited higher resistance than a susceptible strain to the neonicotinoids imidacloprid, clothianidin, acetamiprid, thiacloprid, and thiamethoxam^5^. In China, *A*. *gossypii* was also reported to be resistant to imidacloprid^6,7^. Therefore, new insecticides should be introduced to control resistant *A*. *gossypii*.

Cycloxaprid, 9-((6-chloropyrid-3-yl)methyl)-4-nitro-8-oxa-10,11-dihydroimidazo-[2,3-a]-bicyclo-[3,2,1]-oct-3-ene, is a novel *cis*-nitromethylene neonicotinoid insecticide^8^. This insecticide contains a unique chemical structure with the nitro substituent in the *cis*-configuration, whereas the nitro is in the *trans*-configuration in all other commercialized neonicotinoids^9^. Cycloxaprid was designed to control a wide range of sap-feeding insect pests that are resistant to imidacloprid and other widely used neonicotinoids^10–12^. Compared with imidacloprid, cycloxaprid exhibits 50-fold higher activity against imidacloprid-resistant brown plant hopper^10,11^, and Cui *et al*. demonstrated that cycloxaprid is an excellent insecticide for the control of imidacloprid-resistant *A*. *gossypii*^7^.

In addition to lethal toxicity, possible sublethal effects must also be considered for a comprehensive understanding of a new insecticide. A sublethal dose of an insecticide does not kill the entire population of an insect but exerts physiological and/or behavioural effects on individuals^13^. Exposure of pests to sublethal concentrations of pesticides is a common phenomenon in agro-ecosystems because pesticides are degraded after initial applications to crops^14^. These sublethal effects may impair fundamental physiological and/or behavioural traits, including biochemical and neurophysiological processes, development rates, longevity, reproduction, immune capacity, sex ratio, and feeding, searching, learning, and oviposition activities^15,16^. These types of impairment undoubtedly have important consequences, including the rapid development of tolerance or resistance and outbreaks of insect pests at the population level of the exposed individuals^17^. Therefore, demographic toxicological analysis of an insecticide and estimating the total effect on a population are crucial when choosing new insecticides for IPM. Life table analysis is a useful tool to study population effects that may be underestimated at the individual level^18,19^. In this study, we employed age-stage life table analysis to assess the sublethal effects of cycloxaprid, with a particular focus on possible transgenerational effects, on *A*. *gossypii*. To achieve a more complete understanding of the response of *A*. *gossypii* to cycloxaprid, we also investigated cycloxaprid sublethal effects on detoxification and target enzymes, namely, P450, GST, carboxylesterase (CarE) and acetylcholinesterase (AChE).

## Results

### Toxicity of cycloxaprid against *A*. *gossypii*

Table 1 presents the LC_20_, LC_50_ and LC_90_ values of cycloxaprid against *A*. *gossypii*. Cycloxaprid was toxic to the tested strain of *A*. *gossypii*, with LC_50_ and LC_90_ values of 2.63 and 15.72 mg a.i. L^−1^, respectively. The LC_20_ of cycloxaprid was estimated to be 0.81 mg a.i. L^−1^, and this concentration was used for the subsequent study of sublethal effects. The mortality of *A*. *gossypii* at the LC_20_ of cycloxaprid was 19.51 ± 1.78% after 24 h.

**Table 1:** LC values of cycloxaprid against *A*. *gossypii*.

Insecticide	*n*	Slope ± SE	LC_20_ (mg a.i. L^−1^) 95% CI	LC_50_ (mg a.i. L^−1^) 95% CI	LC_90_ (mg a.i. L^−1^) 95% CI	*P*
Cycloxaprid	2910	1.65 ± 0.21	0.81 (0.51–1.30)	2.63 (1.82–3.78)	15.72 (8.79–28.11)	0.0164

95% CI, 95% confidence interval; SE, standard error.

### Transgenerational sublethal effects of cycloxaprid on F1 generation individuals

Due to its degradation by various factors, the LC_20_ was chosen to mimic the lower concentrations of cycloxaprid that may occur in the field following initial insecticide application^15,20^. In our study, we investigated the sublethal effects of the cycloxaprid LC_20_ on the development time, longevity and fecundity of *A*. *gossypii* in the F1 generation (Table 2). The adult preoviposition period (APOP) decreased significantly in cycloxaprid-treated *A*. *gossypii*. Although cycloxaprid reduced the developmental time of each instar (N1, N2, N3, N4) and the adult, no significant differences were found between control and cycloxaprid-treated *A*. *gossypii*. Moreover, the sublethal concentration of cycloxaprid did not significantly affect the pre-adult and N1-adult periods, mean longevity or the total preoviposition period (TPOP). F1-generation adults exposed to the LC_20_ of cycloxaprid produced more offspring than control adults, though the difference was not significant. The transgenerational effects of sublethal cycloxaprid on the development of population dynamics were estimated using bootstrap methods based on the life table. The *r*_*i*_, *λ*, *R*_0_, and *T* were calculated and analysed (Table 3); however, no significant differences were observed in the cycloxaprid LC_20_ exposed group compared with the control.

**Table 2:** Developmental time and fecundity of the F1-generation *A*. *gossypii* when parents (F0) were treated with the LC_20_ of cycloxaprid.

Stage	Control			Cycloxaprid			*P*
	*n*	Mean	SE	*n*	Mean	SE	
First-instar nymph (N1) (d)	74	1.80	0.09	74	1.73	0.08	0.53
Second-instar nymph (N2) (d)	74	1.49	0.08	71	1.46	0.08	0.79
Third-instar nymph (N3) (d)	73	1.24	0.07	69	1.22	0.06	0.86
Fourth-instar nymph (N4) (d)	71	1.07	0.06	67	1.05	0.06	0.83
Adult (d)	71	13.74	1.03	67	13.49	0.78	0.84
Preadult (d)	71	5.51	0.11	67	5.46	0.1	0.70
N1-Adult (d)	71	19.25	1.06	67	18.94	0.8	0.81
Mean longevity (d)	87	16.32	1.1	85	15.68	0.94	0.65
APOP (d)	69	**0**.**25**	0.07	67	**0**.**09**	0.03	**<0**.**05**
TPOP (d)	69	5.71	0.11	67	5.54	0.1	0.26
Fecundity (offspring/adult)	71	16.85	1.32	67	17.24	1.02	0.81

APOP: adult pre-oviposition period, TPOP: total pre-oviposition period, *n*: number. Bold text indicates a significant difference between the control and cycloxaprid groups (*P* < 0.05, Student’s *t*-test).

**Table 3:** Transgenerational effects of cycloxaprid on population parameters of the F1 generation of *A*. *gossypii*.

Parameters	Control		Cycloxaprid		*P*
	Mean	SE	Mean	SE	
*r*_*i*_ (d^−1^)	0.2703	0.0114	0.2795	0.0109	0.56
*λ* (d^−1^)	1.3104	0.0149	1.3225	0.0144	0.56
*Ro* (offspring/individual)	13.75	1.28	13.59	1.1	0.92
*T* (d)	9.69	0.26	9.33	0.25	0.32
GRR (offspring/individual)	26.25	1.57	24.25	1.704	0.39

*r*_*i*_: intrinsic rate of increase (d^−1^), *λ*: finite rate of increase (d^−1^), *R*_0_: net reproductive rate (offspring/individual), *T*: mean generation time (d). GRR: gross reproduction rate (offspring/individual). The standard error (SE) of the mean values was estimated using 10,000 bootstrap replications.

**Figure 1:**
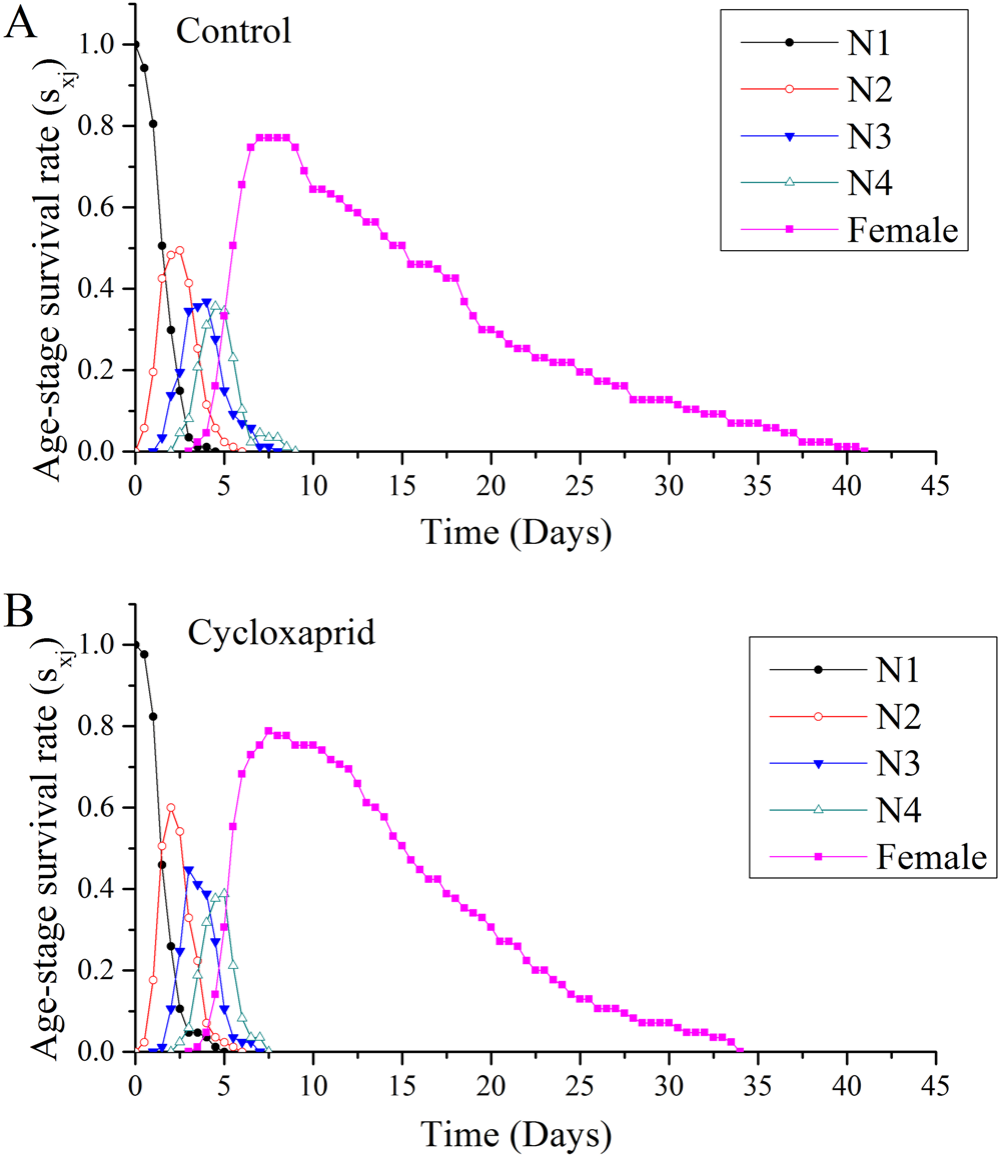
Age-stage survival rate (*s*_*xj*_) of the F1-generation *A*. *gossypii*.

Age-stage survival rate (*s*_*xj*_) curves represent the probability that a newborn nymph will survive to age *x* and stage *j* (Fig. 1). Because of the variable developmental rates of individuals, overlaps among stages were obviously observed in control and cycloxaprid-treated *A*. *gossypii*. The relative numbers of second-instar (N2), third-instar (N3) and fourth-instar (N4) nymphs were higher in the cycloxaprid treated group than in the control group. Additionally, the probability that a newborn nymph survived to an adult was 0.79 for the cycloxaprid-treated group, compared with only 0.77 for the control group.

**Figure 2:**
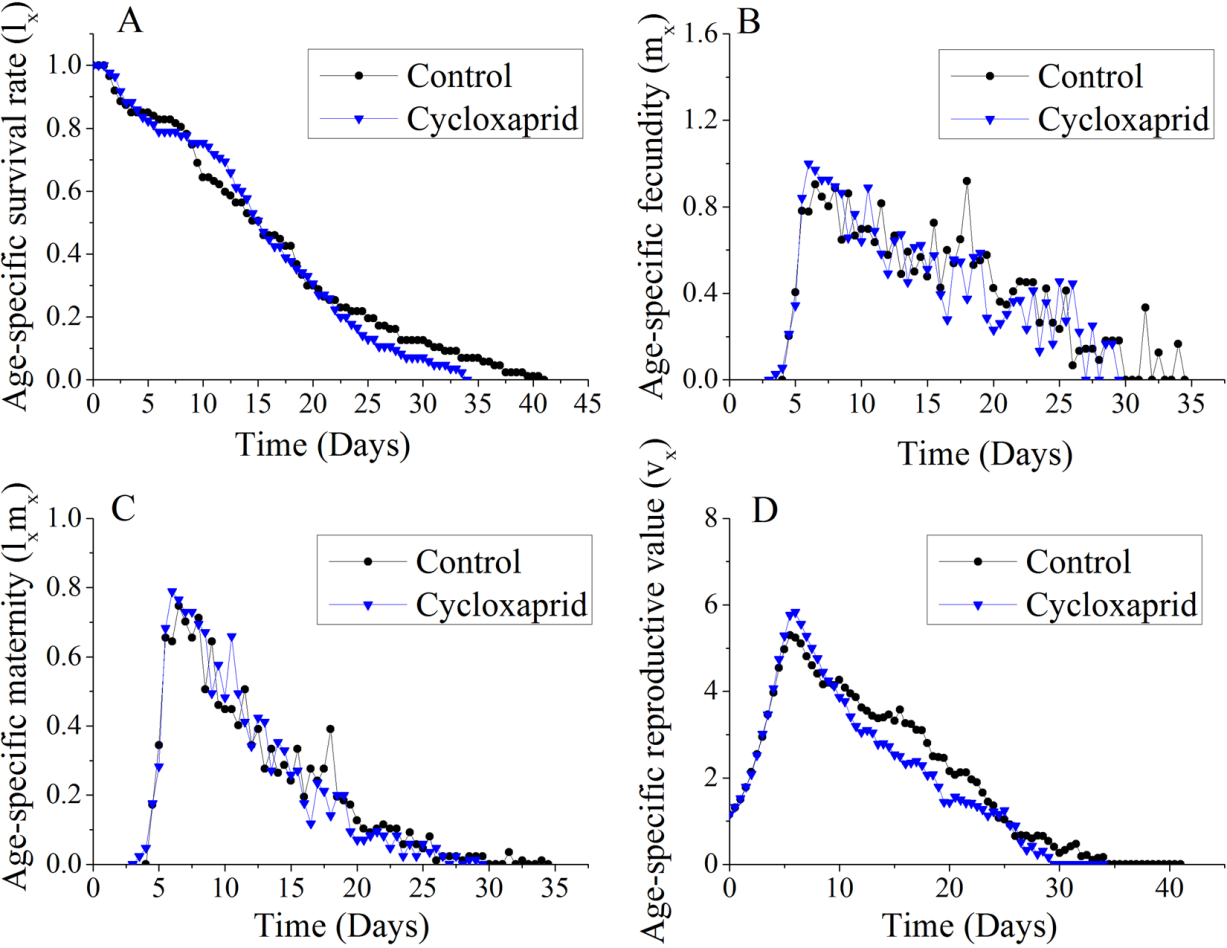
Age-specific survival rate (*l*_*x*_), age-specific fecundity (*m*_*x*_), *l*_*x*_*m*_*x*_ and age-specific reproductive value (*v*_*x*_) of the F1-generation *A*. *gossypii*.

Age-specific survival rate (*l*_*x*_) curve shows the probability that a newborn nymph will survive to age *x*, and *lx* gives a simplified overview of the survival history. As shown in Fig. 2A, *A*. *gossypii* could successfully survive and reproduce when treated with a sublethal concentration of cycloxaprid. However, the *lx* for cycloxaprid-treated *A*. *gossypii* was considerably lower than that of the control after 22 days. Based on the curve *m*_*x*_, the highest age-specific fecundity peak of the control *A*. *gossypii* (0.92 offspring/12 h) occurred at the age of 18.0 d. In contrast, the cycloxaprid LC_20_-treated group responded differently, with a high peak at the age of 6.0 days (1.0 offspring/12 h; Fig. 2B). Dependent on both *l*_*x*_ and *m*_*x*_, the maximal *l*_*x*_*m*_*x*_ values were 0.788 and 0.747 offspring for the cycloxaprid-treated and control groups, respectively (Fig. 2C). The age-specific reproductive value (*v*_*x*_) of the cycloxaprid group was lower than that of the control group in the adult stage after 9.5 days, but the maximum *v*_*x*_ value (5.83 at the age of 6 days) was higher than that of the control (5.29 at the age of 5.5 days) (Fig. 2D).

**Figure 3:**
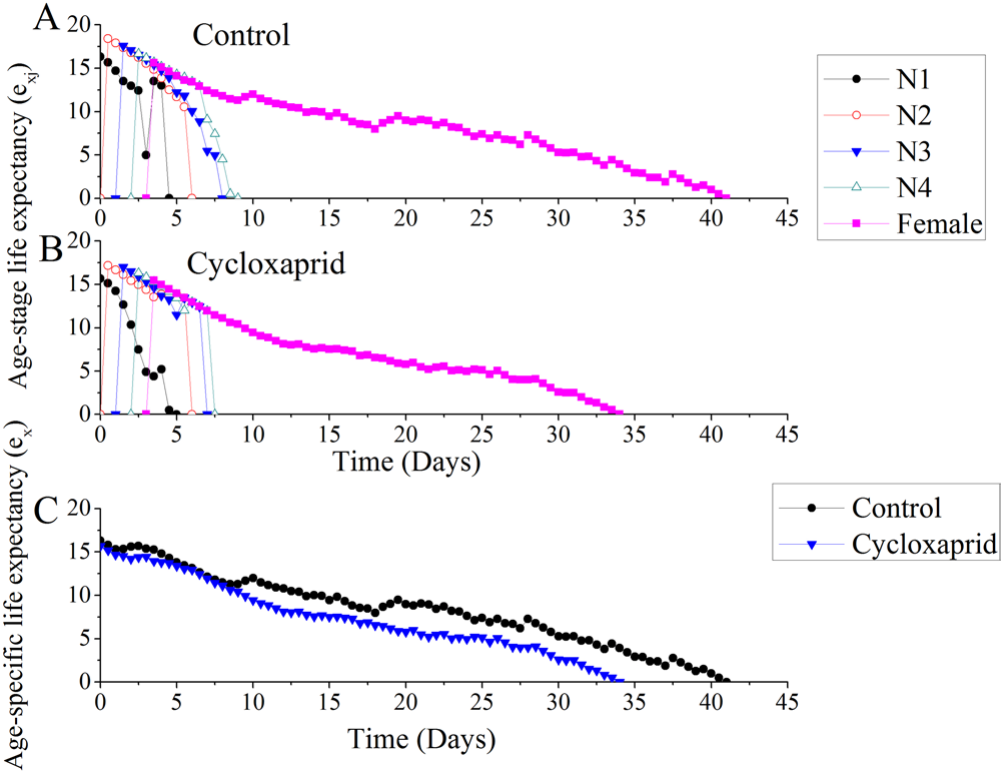
Age-stage life expectancy (*e*_*xj*_) and age-specific life expectancy (*e*_*x*_) for the F1-generation *A*. *gossypii*.

Age-stage life expectancy (*e*_*xj*_) is the time that an individual of age *x* and stage *y* is expected to live, and life expectancy decreased as age increased (Fig. 3). The life expectancy of first-instar (N1), second-instar (N2) and third-instar (N3) nymphs was 15.68, 17.19 and 16.98 days for cycloxaprid-treated *A*. *gossypii*, respectively, whereas these values were as high as 16.32, 18.41 and 17.58 days, respectively, for the control *A*. *gossypii*. Moreover, as indicated in Fig. 3C, cycloxaprid reduced the life expectancy of F1-generation *A*. *gossypii*.

### The sublethal effect of cycloxaprid on *A*. *gossypii* detoxification and target enzymes

**Figure 4:**
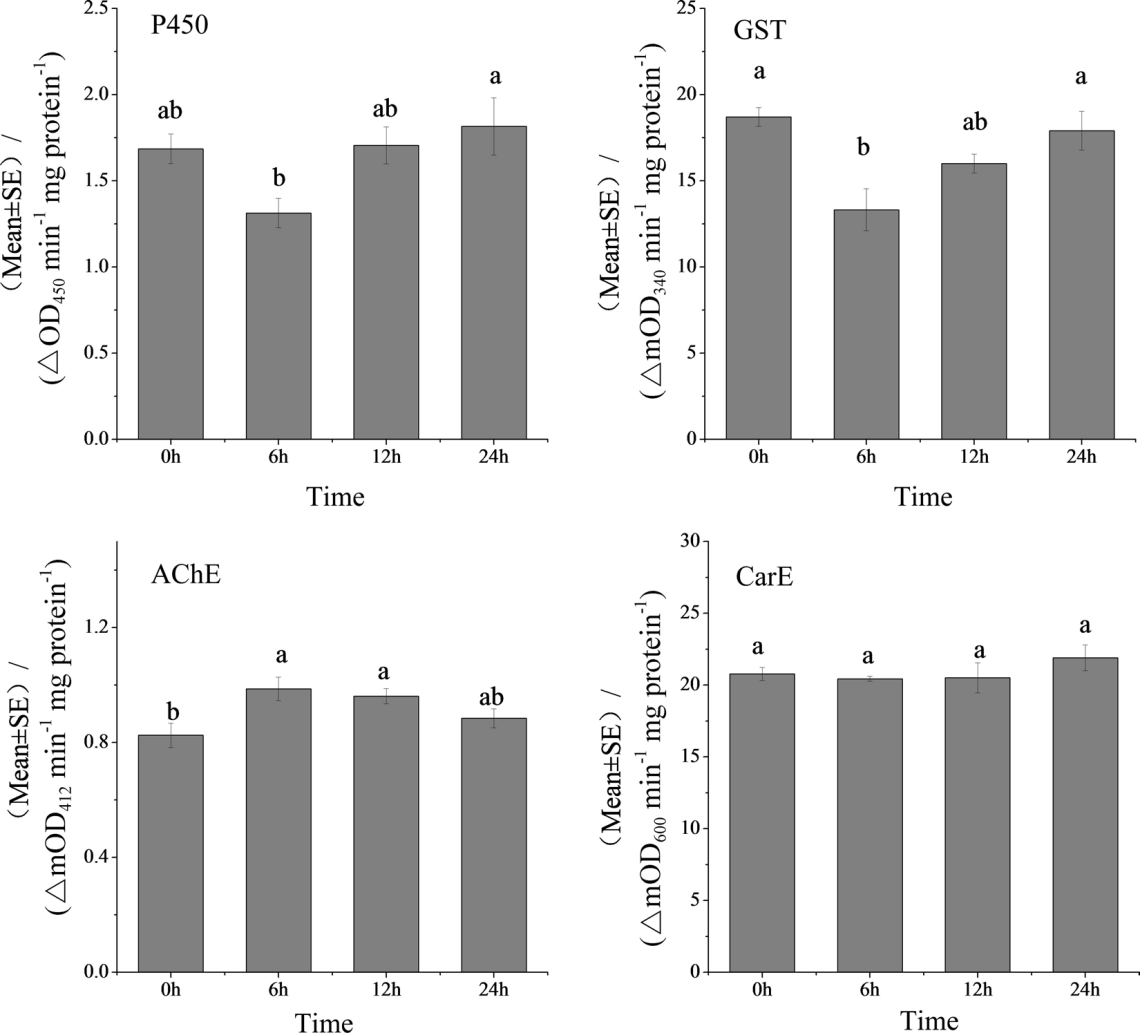
The activity of P450, CarE, GST and AChE of *A*. *gossypii* treated with the LC_20_ of cycloxaprid. The results are presented as mean ± SE. The same letters above the bars indicate no significant difference at *P* < 0.05.

The activities of detoxification enzymes such as P450, CarE, and GST, as well as the target enzyme, AChE in *A*. *gossypii* treated with a sublethal concentration of cycloxaprid are presented in Fig. 4. No significant difference (*P* = 0.49) in CarE activity was detected between cycloxaprid-treated *A*. *gossypii* and the untreated control. However, cycloxaprid significantly stimulated AChE enzyme activity (*P* = 0.049) at 6 and 12 h after treatment, with 19.5% and 16.4% higher activity measured, respectively. The responses of P450 and GST activities to cycloxaprid exposure were similar, decreasing by 10.1% (*P* = 0.079) and 28.8% (*P* = 0.013), respectively, after 6 h; however, activities tended to increase to the same levels as the control thereafter.

## Discussion

Previous studies demonstrate that cycloxaprid is effective for the control of *A*. *gossypii* in the field^7^. Additionally, cycloxaprid shows highly selective activity between *A*. *gossypii* and its predominant natural enemies, *Harmonia axyridis* (Pallas) (Coleoptera: Coccinellidae) and *Chrysoperla sinica* Tjeder (Neuroptera: Chrysopidae)^7^. Based on these results, cycloxaprid can serve as an alternative to other insecticides for the control of *A*. *gossypii* and may be widely used in agriculture in the coming year. However, because of variable distribution and continuous degradation, insect pests will frequently be exposed to low concentrations of cycloxaprid in the field, resulting in potential sublethal effects, e.g., modifications of feeding behaviour, survival rate, development time, fecundity and resistance development^21^. These sublethal effects by insecticides can negatively or positively affect the fitness of insect pests. This biphasic phenomenon, characterized by low-dose stimulation and high-dose inhibition following exposure to stress, is known as hormesis^22^. In addition to stimulating life history traits, hormesis may lead to insect pest outbreaks or augment the development of insecticide tolerance or resistance^23,24^; such events occur when exposure to low doses of a pesticide induces production of enzymes that detoxify the compound or increases mutations that confer resistance^22^. For example, sublethal concentrations of imidacloprid increase reproduction, alter expression of detoxification genes (cytochrome P450-CYP6CY3, E4-esterase and Hsp60 genes), and prime *Myzus persicae* (Sulzer) (Hemiptera: Aphididae) for subsequent stress^22^. Additionally, Gressel found that low pesticide rates may accelerate the evolution of resistance by increasing mutation frequencies^25^. Therefore, the sublethal effects and risks of cycloxaprid application must be determined.

The *r*_*i*_ shows the ability of a population to increase logarithmically in an unlimited environment, and this parameter has been proposed as a more reliable measurement of insecticidal toxic effects than estimates of lethal concentrations^26^. According to our findings, sublethal exposure of parent *A*. *gossypii* slightly increased *r*_*i*_ and decreased the developmental time of nymphs and adults in the offspring. However, the differences were not significant when compared with control *A*. *gossypii*. These results indicate that cycloxaprid at the LC_20_ sublethal concentration would not induce hormesis in *A*. *gossypii*. Similarly, it was also reported that cycloxaprid at LC_10_ and LC_40_ sublethal concentrations had no significant effect on the *T*, *r*_*i*_, *λ* or doubling time (*DT*) of *A*. *gossypii*^27^. These results suggest that insecticide-induced resurgence might not occur after exposure of adult *A*. *gossypii* to low sublethal concentrations of cycloxaprid. Yuan *et al*. documented that the longevity of *A*. *gossypii* treated with the LC_40_ of cycloxaprid was lower than that of control aphids and those exposed to the LC_10_ concentration^27^. This result was consistent with a report that *A*. *gossypii* longevity was reduced by 12 days, when adults were treated with a sublethal concentration of imidacloprid^28^. Moreover, sublethal effects of cycloxaprid have been documented in other important insect pests. For example, a sublethal concentration of cycloxaprid impaired *Sitobion avenae* (Fabricius) (Hemiptera: Aphididae) phloem ingestion and thereby reduced the weight of aphids^11^. Additionally, cycloxaprid at the LC_25_ concentration induced sublethal effects in adult *Bemisia tabaci* (Gennadius) (Hemiptera: Aleyrodidae) by prolonging developmental periods and decreasing survival rates among all larval instar, pseudopupal and adult stages and also significantly shortened the oviposition period of females and decreased their fecundity^29^. Cycloxaprid at low concentrations also strongly reduced *Apolygus lucorum* (Meyer-Dür) (Hemiptera: Miridae) adult longevity, decreased female fecundity, and prolonged the pre-oviposition period while shortening the oviposition period^12^.

Responses of enzyme activity can be used as “biomarkers” to assess sublethal contamination in invertebrates and vertebrates^30^. Thus, the determination of detoxification enzyme activities could add valuable information to the overall understanding of the effect of cycloxaprid on *A*. *gossypii* populations. In this *in vivo* study, four enzyme systems (i.e., P450, GST, CarE and AChE) were examined as possible biomarkers for cycloxaprid-treated *A*. *gossypii*. Cytochrome P450 monooxygenases are the largest and most functionally diverse class of insect detoxification enzymes; members of the CYP3 clade are implicated in the oxidative detoxification of synthetic insecticides^31^. Similar to P450s, carboxyl/cholinesterases (CCEs) function broadly in xenobiotic detoxification, and clades A to C are involved in insecticide detoxification. Insect CCEs can hydrolyse both organophosphates and other synthetic insecticides^32^. GST enzymes function by conjugating xenobiotics and endogenously activating compounds to the thiol group of reduced glutathione, thereby targeting them for more rapid excretion or degradation^33^. In insects, GSTs are associated with resistance to insecticides, including spinosad, diazinon and nitenpyram^34^. Our results showed that CarE activity in *A*. *gossypii* was not induced by cycloxaprid exposure, suggesting that CarE is not the primary factor affecting cycloxaprid detoxification and resistance in *A*. *gossypii*. Conversely, the activity of AChE was significantly induced by the sublethal concentration of cycloxaprid, which might be the result of increased expression of the enzyme at the transcriptional or translational level. In contrast, cycloxaprid inhibited the activities of P450 and GST after 6 h of exposure, similar to the findings of Yin *et al*.^35^, Obear *et al*.^36^ and Rumpf *et al*.^30^. However, this inhibition was reversed and the activities of P450 and GST recovered over time. The recovery of enzyme activities might be associated with *de novo* synthesis, as assessed by Fossi *et al*.^37^. Additionally, inhibition of P450 and GST activities might decrease the metabolic processing of cycloxaprid; thus, cycloxaprid readily exhibits high efficacy against *A*. *gossypii*.

In this study, only the sublethal effect of cycloxaprid on the first generation of *A*. *gossypii* was investigated, without an evaluation of continuous insecticide exposure. Therefore, further study is required to determine cycloxaprid-induced multigenerational hormesis in *A*. *gossypii* and studies using different sublethal concentrations are required. Additionally, exposure of field populations may provide a more comprehensive evaluation of putative hormesis responses of *A*. *gossypii* to cycloxaprid. With the aim of establishing an optimized IPM strategy, the results of the present study under laboratory conditions justify the importance of assessing the sublethal effects of cycloxaprid on *A*. *gossypii* populations in the field.

## Methods

### Insects and insecticides

The laboratory population of cotton aphid (*A*. *gossypii*) used in this study was originally collected from cotton in Xinjiang Province, China; the population has been maintained in our laboratory without exposure to any insecticides since June 2015. *A*. *gossypii* individuals were reared on cotton plants (Zhongzhi 8) and maintained under controlled conditions at 25 ± 2 °C, 70 ± 20% relative humidity (RH) and a 14:10 h light:dark photoperiod.

Cycloxaprid (97%) was obtained from East China University of Science and Technology (Shanghai, China). Dimethylsulfoxide (DMSO) and triton X-100 were purchased from Beijing Chemical Reagent Co., Ltd.

### Toxicity of cycloxaprid against *A*. *gossypii*

The toxicity of cycloxaprid against *A*. *gossypii* under laboratory conditions was evaluated using a previously described leaf-dipping method^7^. The stock solution of cycloxaprid (10,000 mg a.i. L^−1^ in DMSO) was diluted to concentrations of 50, 20, 10, 5, 2, 1, 0.5 and 0.1 mg a.i. L^−1^ using an aqueous solution of 0.05% (w/v) triton X-100. Bioassays consisted of four replicates for each concentration. Individual cotton leaves infested with approximately fifty mixed-age *A*. *gossypii* were dipped in the cycloxaprid solutions for 3 sec and dried on tissue paper. Afterwards, individual leaves were transferred to 90 mm petri dishes containing water-moistened filter paper. Each petri dish was covered with a perforated lid with fine mesh to provide ventilation; the dishes were then stored in an incubator at 25 ± 2 °C, 70 ± 20% RH and a 14:10 h light:dark photoperiod for 24 h until mortality was assessed. Control aphids were treated with distilled water containing 0.05% triton X-100 and DMSO; the mortality of the control samples was less than 10%. The sublethal concentration value (LC_20_), lethal concentration values (LC_50_ and LC_90_) and their 95% confidence intervals (CIs) were calculated using statistical software DPS 7.05 (Refine Information Tech. Co. Ltd, Hangzhou, China). The LC_20_ concentration was used for subsequent experiments evaluating the sublethal effects of cycloxaprid.

### Sublethal effects of cycloxaprid on the F1 generation of *A*. *gossypii*

The LC_20_ concentration obtained from the previous bioassay was used to evaluate the sublethal effects of cycloxaprid on *A*. *gossypii*. Cycloxaprid was prepared in DMSO and diluted to the LC_20_ with distilled water containing 0.05% triton X-100. *A*. *gossypii* dipped in cycloxaprid solution for 3 sec were used as the treatment group, control aphids were treated with aqueous solution of DMSO and 0.05% triton X-100. When dry, the leaves were transferred to 90 mm petri dishes containing water-moistened filter paper and covered with a perforated lid. Mortality was calculated at 24 h after treatment. Surviving adult apterous aphids were gently moved into a separate glass dish containing fresh cotton leaf discs without any insecticide. The offspring produced by these F0 adults on the second day were collected and used as the F1 generation in this life table experiment. At least 150 neonate nymphs of each group were observed individually. The leaf discs placed on agar beds (2% agar) were replaced every 3 days during the experiments. Population parameters, including the developmental time of every stage, survival, the oviposition period, longevity, and the number of progeny produced per female were recorded daily. Newly born nymphs were counted and removed each day. The longevity of the aphids and the number of nymphs produced per female were recorded until the adult was dead. These data were then used to establish the age-stage, two-sex life table. The *l*_*x*_, *s*_*xj*_, *v*_*xj*_, APOP, TPOP, *r*_*i*_, *λ*, *R*_0_ and *T* were calculated. Survival rate, fecundity, and reproductive value curves were constructed using Origin 8.0 software (OriginLab Corporation, Northampton, USA).

### Detoxification enzyme assays

#### Preparation of *A*. *gossypii* homogenate

*A*. *gossypii* aphids were treated with cycloxaprid at the LC_20_ concentration and collected at 0, 6, 12 and 24 h after exposure. *A*. *gossypii* samples (10 mg) were homogenized on ice in 2 mL of 0.04 M phosphate buffer, pH 7.0 (CarE assay), 66 mM phosphate buffer, pH 7.0 (GST assay), 0.1 M phosphate buffer, pH 7.4 (AChE assay) or 0.1 M phosphate buffer, pH 7.6, containing 1 mM ethylenediamine tetraacetic acid (EDTA), 1 mM dithiothreitol (DTT), 1 mM N-phenylthiourea (PTU) and 1 mM phenylmethylsulfonyl fluoride (PMSF) (P450 assay). The homogenate was centrifuged at 10,000 rpm for 15 min at 4 °C, and the supernatant was collected as the enzyme source. The protein concentration of the enzyme source was determined according to the method of Bradford^38^, using bovine serum albumin as the standard.

### CarE ahssay

CarE activity was measured using α-naphthyl acetate as a substrate according to the method described by Van Asperen^39^, with slight modification. Five millilitres of a substrate solution containing 0.3 mM α-naphthyl acetate (α-NA) and 0.1 mM physostigmine (an inhibitor of acetylcholinesterase) was prepared, followed by the addition of approximately 0–0.5 mL of enzyme source (diluted 20-fold) and approximately 1–0.5 mL of phosphate-buffered saline (PBS: 0.04 M, pH 7.0). The mixture was incubated with shaking for 30 min at 30 °C. The reaction was stopped by the addition of 1 mL of distilled water containing 2.9 mg of fast blue B salt and 35.7 mg of sodium dodecyl sulfate (SDS). Absorbance at 600 nm was measured after 30 min using a Synergy HT multi-mode microplate reader (BioTek, Winooski, VT). The results are expressed as ΔmOD_600_ min^−1^ mg protein^−1^. At least three replicates of enzyme sources were tested with 5 individuals for each replicate.

### GST assay

GST activity was measured using 2,4-dinitrochlorobenzene (CDNB) as the substrate^40^. The enzyme solution (0.2 mL) was incubated with CDNB (0.1 mL, 30 mM), glutathione (GSH: 0.3 mL, 50 mM) and PBS (2.4 mL, 66 mM, pH 7.0). Enzyme activity was measured using a Synergy HT multi-mode microplate reader at 340 nm and 27 °C using the kinetic mode for 5 min. The results are expressed as ΔmOD_340_ min^−1^ mg protein^−1^. Three replicates of enzyme sources were tested with 5 individuals for each replicate.

### P450 assay

Insect functional oxidase ELISA kit was used to assay P450 levels in the samples. The enzyme sources were transferred to the microlon ELISA plates in accordance with the manufacturer’s instructions. ODs were measured at 450 nm using a Synergy HT multi-mode microplate reader, and concentrations were calculated by comparing the ODs to the standard curve. The results are expressed as ΔOD_450_ min^−1^ mg protein^−1^. Three replicates of enzyme sources were tested.

### AChE assay

AChE activity was measured using acetylthiocholine iodide as substrate by the method of Ellman^41^. A solution of 0.05 mL of 75 mM acetylthiocholine iodide and 0.1 mL of 0.1 M dithionitrobenzoic acid (DTNB) was prepared, followed by the addition of approximately 0–0.5 mL of enzyme and approximately 2.1–2.6 mL of PBS (0.1 M, pH 7.4). The mixture was incubated with shaking for 15 min at 27 °C. The reaction was stopped by the addition of 0.5 mL of 1 mM physostigmine. Absorbance at 412 nm was measured using a Synergy HT multi-mode microplate reader. The results are expressed as ΔmOD_412_ min^−1^ mg protein^−1^. Three replicates of enzyme sources were tested with 5 individuals for each replicate.

### Statistical analysis

The data for the life history of *A*. *gossypii* individuals were analysed according to the age-stage, two-sex life table using the TWOSEX-MSChart computer program^42^. The means and standard errors of survival, longevity and fecundity were assessed with bootstrapping method^43^ (10,000 bootstrap replications) in the TWOSEX-MSChart program. The differences of data were statistically analysed using one-way analysis of variance (ANOVA) followed by Fisher’s LSD tests and Student’s *t*-tests (α = 0.05) in SPSS 13.0 statistical software package (SPSS, Inc., Chicago, IL, USA).

### Data availability statement

No datasets were generated or analysed during the current study.
